# Cryptochrome 2 from *Lilium × formolongi* Regulates Photoperiodic Flowering in Transgenic *Arabidopsis thaliana*

**DOI:** 10.3390/ijms222312929

**Published:** 2021-11-29

**Authors:** Xiao-Mei Wu, Zheng-Min Yang, Lin-Hao Yang, Ji-Ren Chen, Hai-Xia Chen, Si-Xiang Zheng, Jian-Guo Zeng, Gui-Xia Jia, Yu-Fan Li

**Affiliations:** 1 Hunan Mid-Subtropical Quality Plant Breeding and Utilization Engineering Technology Research Center, College of Horticulture, Hunan Agriculture University, Changsha 410128, China; wuxiaomei@stu.hunau.edu.cn (X.-M.W.); yangzm1125@163.com (Z.-M.Y.); y123l45@163.com (L.-H.Y.); bjfucjr@163.com (J.-R.C.); chenhaixia@hunau.edu.cn (H.-X.C.); 2 Institute of Agriculture Environment and Agro Ecology, Hunan Academy of Agriculture Sciences, Changsha 410125, China; zhengqianlian@163.com; 3 National and Local Union Engineering Research Center of Veterinary Herbal Medicine Resource and Initiative, Hunan Key Laboratory of Traditional Chinese Veterinary Medicine, College of Veterinary Medicine, Hunan Agricultural University, Changsha 410125, China; zengjianguo@hunau.edu.cn; 4 National Engineering Research Center for Floriculture, Beijing Key Laboratory of Ornamental Plants Germplasm Innovation & Molecular Breeding, Beijing Laboratory of Urban and Rural Ecological Environment and College of Landscape Architecture, Beijing Forestry University, Beijing 100083, China

**Keywords:** cryptochrome, *Lilium × formolongi*, photoperiodic flowering

## Abstract

The photoperiodic flowering pathway is essential for plant reproduction. As blue and ultraviolet-A light receptors, cryptochromes play an important role in the photoperiodic regulation of flowering. *Lilium × formolongi* is an important cut flower that flowers within a year after seed propagation. Floral induction is highly sensitive to photoperiod. In this study, we isolated the *CRYPTOCHROME2* gene (*LfCRY2*) from *L. × formolongi*. The predicted LfCRY2 protein was highly homologous to other CRY2 proteins. The transcription of *LfCRY2* was induced by blue light. *LfCRY2* exhibits its highest diurnal expression during the floral induction stage under both long-day and short-day photoperiods. Overexpression of *LfCRY2* in *Arabidopsis thaliana* promoted flowering under long days but not short days, and inhibited hypocotyl elongation under blue light. Furthermore, LfCRY2 was located in the nucleus and could interact with *L. × formolongi* CONSTANS-like 9 (LfCOL9) and *A. thaliana* CRY-interacting basic-helix-loop-helix 1 (AtCIB1) in both yeast and onion cells, which supports the hypothesis that *LfCRY2* hastens the floral transition via the CIB1-CO pathway in a manner similar to *AtCRY2*. These results provide evidence that *LfCRY2* plays a vital role in promoting flowering under long days in *L. × formolongi*.

## 1. Introduction

The transition from vegetative to reproductive growth is important for flowering plants. The initiation of this transition is controlled by both developmental and environment signals. One important signal that regulates floral induction is daylight length (photoperiod) [1]. The photoperiodic control of floral induction is called the photoperiodic flowering pathway [2]. Plants have evolved multiple photoreceptors that can perceive light of various wavelengths. Phytochromes can sense red to far-red light (600–750 nm) [3], while blue and ultraviolet (UV)-A (320–500 nm) photoreceptors include cryptochromes (CRYs), phototropins, ZEITLUPE (ZTL), flavin binding, Kelch repeat, F-box 1 (FKF1), and LOV, Kelch, protein 2 (LKP2) [4,5]. UV-B resistance 8 (UVR8) can sense UV-B light (280–320 nm) [6]. 

CRYs are flavin-containing proteins that mediate light-sensitive responses [7,8] in bacteria, fruit flies (*Drosophila melanogaster*), plants, and mammals [9]. Plant CRYs are photolyase-related blue light receptors involved in the regulation of photomorphogenesis and blue light (BL) induction of stomatal opening and de-etiolation [10,11,12]. Moreover, CRYs have been shown to act as components of the circadian clock and participate in photoperiodic flowering [8]. Plants have at least two types of CRYs: CRY1 and CRY2 [12,13]. In *Arabidopsis*, the main function of CRY1 is to regulate de-etiolation under blue light [12], whereas CRY2 mainly mediates photoperiodic flowering [13]. CRYs are typically comprised of a nitrogen (N)-terminal photolyase homologous region (PHR) domain that includes regions required to bind to the two chromophores, flavin adenine dinucleotide (FAD) and methenyltetrahydrofolate (MTHF), and a CRY-specific C-terminal extension domain (CCE) that is absent in photolyase and has a different sequence than any known protein domain [14,15]. Although the CCE domains of CRY1 and CRY2 are significantly different, they both play an important role in light signal transmission [16]. The CCE domain is necessary for interactions with other proteins [15,17]. The *Arabidopsis* CRY1 protein is located in both the nucleus and cytosol and is stable under blue light, whereas CRY2 accumulates exclusively in the nucleus, is unstable, are rapidly phosphorylated and degraded under blue light [18,19]. 

It has been suggested that CRY2 is the predominant photoreceptor participating in the photoperiodic regulation of flowering in *Arabidopsis* [13], whereas CRY1 plays a minor role in the floral induction stage [20]. CONSTANS (CO) and FLOWERING LOCUS T (FT) are among the most important regulators of floral induction in the photoperiodic flowering pathway [21]. CO is a critical positive transcription factor of flowering by activating *FT* mRNA expression [22,23]. FT protein acts as a long-distance signal by migrating from the leaves to the shoot apex to activate the transcription of floral meristem identity genes and induce flowering [24]. *Arabidopsis CRY2* mediate photoperiodic flowering by interacting with a variety of downstream components to regulate the expression of *CO* and *FT*. For example, CRY2, together with SUPPRESSOR OF PHYA-105 1 (SPA1), suppresses the CONSTITUTIVE PHOTOMORPHOGENIC 1 (COP1)-dependent degradation of CO, leading to *FT* transcription and promoting photoperiodic flowering [15,25,26]. COP1 targets CO and is a major negative regulator of both photomorphogenesis and floral induction [27]. Another pivotal transcription factor in *CRY2*-mediated photoperiodic flowering is the CRY-interacting basic-helix-loop-helix 1 (CIB1) transcription factor. CIB1 promotes flowering in a *CRY*-dependent manner by activating the transcription of *CRY2* and *FT* [28]. Furthermore, CIB1 physically interacts with CO and they form a protein complex with CRY2 in response to blue light to activate *FT* transcription [29]. Additionally, CRY2 interacts with the transcription factors TARGET OF EAT 1 (TOE1) and TOE2 directly to promote the dissociation of TOE1 and TOE2 from CO, resulting in the alleviation of their inhibition of CO transcriptional activity, accelerating floral induction [30]. 

The function of CRYs in the promotion of flowering in other angiosperms, including rice (*Oryza sativa CRY2)*, soybean (*Glycine max CRY1a)*, sorghum (*Sorghum bicolor CRY1b)*, and chrysanthemum (*Chrysanthemum lavandulifolium CRY1a* and *CRY1b*)*,* is fundamentally similar to that of CRYs in *Arabidopsis*, [31,32,33,34]. For example, in rice, the flowering time of *Oryza sativa CRY2-RNAi* transgenic lines was significantly delayed under both long days (LDs) and short days (SDs), suggesting that CRY2 plays an important role in the photoperiodic regulation of flowering [31]. Unlike in *Arabidopsis*, *Glycine max CRY1* is the major flowering regulator in soybeans. *Glycine max* CRY1 promotes floral induction and exhibits a circadian rhythm in protein levels under different photoperiods [32]. Among ornamental plants, only *Chrysanthemum lavandulifolium* has two *ClCRY1*s genes. *ClCRY1a* and *ClCRY1b* promote flowering during both SDs and LDs in *Arabidopsis* [34]. In addition to promoting photoperiodic flowering, *CRY2* inhibits hypocotyl elongation under low irradiances of blue light [35]. In *Arabidopsis*, overexpression of the CRY2 protein in a *cry1 cry2* double mutant can lead to long-hypocotyl and late-flowering phenotypes [36].

*Lilium × formolongi* is an important cut flower that can flower within a year of seed propagation and does not require cold exposure (vernalization) to induce flowering. Understanding the molecular genetic mechanisms of photoperiodic flowering is very important for understanding the annual flowering of this cultivar. As with *Arabidopsis*, *L. × formolongi* is sensitive to photoperiod and we have confirmed it to be a facultative LD plant [37]. Additionally, we analyzed the function of the critical photoperiodic flowering activator *Lilium CONSTANS-like 9 (LfCOL9)* [38]. However, the molecular mechanisms of photoperiodic flowering in lilies are mostly unknown. In this study, we identified and isolated a *CRY2* gene from *L. × formolongi*. The role of *LfCRY2* in photoperiodic flowering was established, and the interactions between LfCRY2 and LfCOL9 and AtCIB1 were confirmed following deep investigation into the action of *LfCRY2*. Our results indicate that *LfCRY2* plays an important role in the photoperiodic regulation of flowering in lilies, providing new insight into the molecular mechanisms regulating this process.

## 2. Results

### 2.1. Cloning and Sequence Analysis of LfCRY2

*LfCRY2* had been identified from the RNA-sequencing libraries of *L. × formolongi* [37]. Specific primers (Table S1) were designed to amplify the encoding sequences of *L. × formolongi* by PCR and then sequenced. *LfCRY2* contained a 1977 base pair (bp) open reading frame (ORF), which encoded a protein of 658 amino acid residues with a calculated molecular mass of 74.1 kDa and a theoretical isoelectric point of 6.49 (accession number MK577786). The predicted LfCRY2 shared a 59.64% sequence identity with *Arabidopsis* CRY2 and an even higher sequence identity with CRY2 orthologs in monocotyledons, such as *Elaeis guineensis* CRY2 (72.89%) and *Oryza sativa* CRY2 (64.15%).

**Figure 1:**
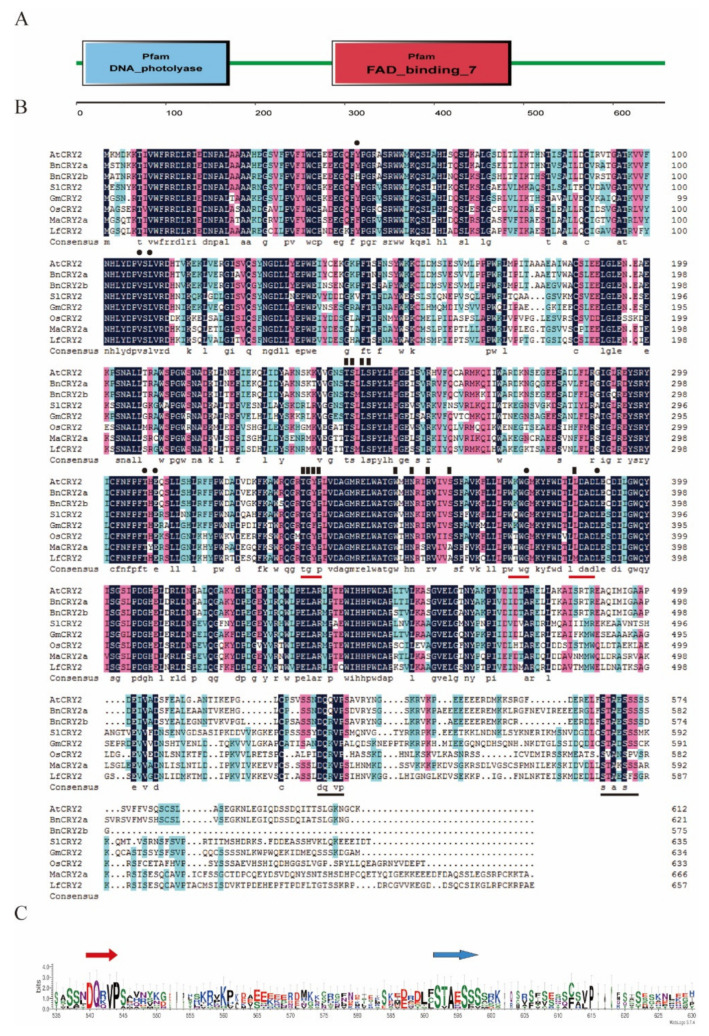
Analysis of LfCRY2 protein sequence. (**A**) Structural domains of the LfCRY2 protein. Analysis of protein sequences by SMART program. (**B**) Amino acid sequence alignment of cryptochrome proteins from *Arabidopsis*, *Brassica napus*, *Solanum lycopersicum*, *Glycine max*, *Oryza sativa*, and *Musa acuminate*. The alignment was constructed using DNAman version 5.2.2 software. Identical residues are highlighted by black boxes. Red lines under the sequences indicate the TGYP, WRWK, and LLDAD motifs. Black lines above the sequences indicate the DQMVP-E/D-STAESS (DAS) domain located in the C-terminal region. Residues that interact with FAD and MTHF are indicated by black rectangles and black dots, respectively. (**C**) DAS domain sequence logos. The sequence alignment of the domains was generated using ClustalX, and conserved motif logos were created using the WebLogo program (http://weblogo.threeplusone.com accessed on 31 October 2021).

All previously described plant CRYs consist of a well-conserved N-terminal PHR superfamily which included the DNA photolyase domain and FAD-binding domain, as well as a shorter, highly variable C-terminal extension (CCE) domain. The deduced amino-acid sequence of LfCRY2 was aligned with those found in other plants (both dicotyledons and monocotyledons), such as *Arabidopsis thaliana*, *Brassica napus*, *Solanum lycopersicum*, *Glycine max*, *Oryza sativa,* and *Musa acuminata*. Similar to other CRY2 orthologs, the amino acid sequence of LfCRY2 contained a well-conserved DNA photolyase domain and FAD-binding domain (Figure 1A), although the length and similarity of the extreme C-terminus among LfCRY2 differed from other orthologs, but the two most prominent motifs (DQXVP and STAESS) in the C-terminal were conserved (Figure 1B,C). In *Arabidopsis* CRYs, the PHR domain was required for binding with the cofactors methenyltetrahydrofolate (MTHF) and chromophore flavin adenine dinucleotide (FAD) [39,40]. All seven amino acids predicted to interact with the MTHF in AtCRY2 were conserved in LfCRY2 (black dots in Figure 1B), while 11 out of 13 amino acids in AtCRY2 known to interact with FAD were conserved in LfCRY2 apart from the serine at position 362, which was replaced with alanine and the leucine at position 385, which was replaced with methionine (black triangles in Figure 1B). Additionally, LfCRY2 included a TGYP motif conserved in all type I photolyases [40]. The amino acid arginine at position 374 was replaced with threonine in the WRWG motif, which was a conserved motif in most CRYs [12]. The leucine at position 385 was replaced with methionine in the LLDAD motif, a conserved region of the FAD-binding domain of CRYs (Figure 1B). The alignment in Figure 1B showed that the overall similarity in the C-terminal extension (CCE domain) was low but the two hallmark motifs were generally conserved in LfCRY2 and other orthologs (Figure 1C). The two motifs are known as the DAS domain, which includes the DQXVP motif (function unknown), and the STAESS motif (replaced by STAESF in LfCRY2) (Figure 1B,C).

### 2.2. Phylogenetic and Amino Acid Similarity Analysis of LfCRY2

**Figure 2:**
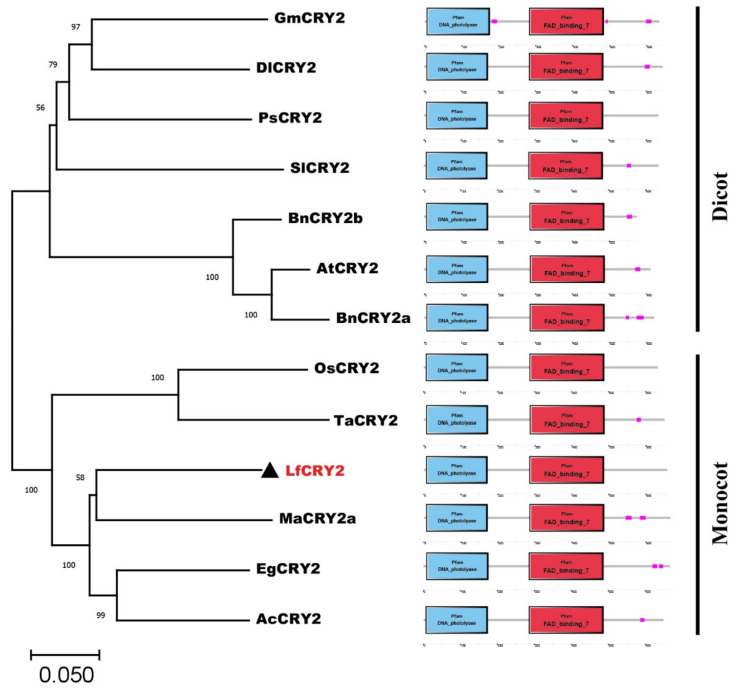
The phylogenetic relationship and conserved domain analysis of CRY2 homologs. CRY2 amino acid sequences from 12 different plant species were acquired from the NCBI database. The phylogenetic tree was generated using the neighbor-joining method. Bootstrap values from 1000 replicates were used to assess the robustness of the tree. The scale indicates the average number of substitutions per site. LfCRY2 is denoted by black triangles. At, *Arabidopsis thaliana*; Bn, *Brassica napus*; Sl, *Solanum lycopersicum*; Ps, *Paeonia suffruticosa*; Gm, *Glycine max*; Dl, *Dimocarpus longan*; Ma, *Musa acuminate*; Ac, *Ananas comosus*; Ta, *Triticum aestivum*; Eg, *Elaeis guineensis*; Os, *Oryza sativa*. The structure diagrams on the right-hand side show the domain structures of the DNA photolyase domain (blue rectangles), and FAD-binding domain (red rectangles) of the CRY2 amino acid sequences.

A phylogenetic analysis of CRY2 orthologs from 12 species was performed using the neighbor-joining method MEGA 11.0. Dicotyledons and monocotyledons were classified into two groups (Figure 2). The CRY2 orthologs from *Arabidopsis*, *Brassica napus* (JF303654 and JF303655) were classified in the dicotyledonous group. *Glycine max* CRY2 (DQ401047) was classified with the orthologs of *Paeonia suffruticosa* (ALB08478), *Solanum lycopersicum* (NM_001247316), and *Dimocarpus longan* (AHZ89699). LfCRY2 was grouped into the monocotyledonous group together with rice and wheat orthologs, but they were in different clades (Figure 2). LfCRY2 was classified with the *Musa acuminate* (APX43204), *Elaeis guineensis* (XP_010920471), and *Ananas comosus* (OAY72788) orthologs and showed the highest homology with *Musa acuminate* CRY2 (Figure 2). These results demonstrated that the phylogenetic tree was consistent with plant evolution. Moreover, in all 12 CRY2 orthologs, the N-terminal DNA photolyase domain and FAD-binding domain were conserved, while the C-terminal CCE domains were short and variable (Figure 2).

### 2.3. Tissue-Specific Expression and Photoperiodic Regulation of CRY2 Gene in L. × formolongi

**Figure 3:**
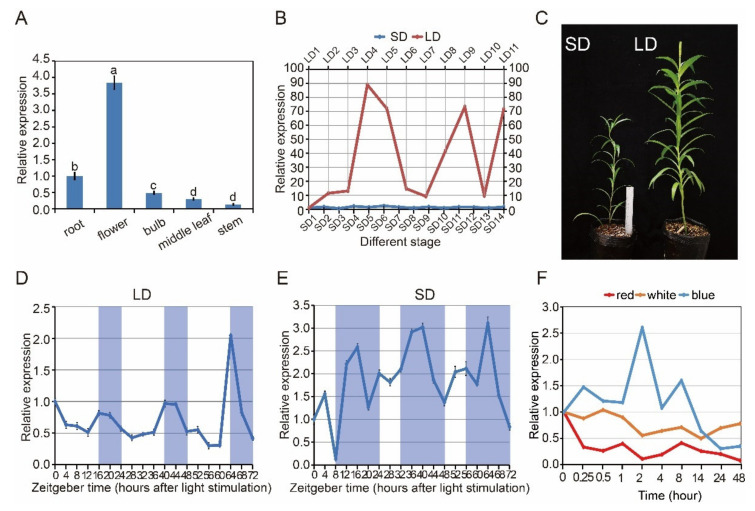
Analysis of LfCRY2 expression using real-time quantitative RT-qPCR. (**A**) LfCRY2 transcript levels in different tissues at the flowering stage under LDs (17.0 µmol m^−2^ s^−1^ white light, light/dark cycle of 16/8 h). (**B**) The stage-specific expression patterns of LfCRY2 in the leaves under different photoperiods. Developmental stages corresponding to each time point are as follows: LD1, 2–3 rosette leaves; LD2, 4–5 rosette leaves; LD3, 6–7 rosette leaves; LD4, 8–9 rosette leaves; LD5, 1–2 internodes (just bolting); LD6, 4–5 internodes; LD7, 7–8 internodes; LD8, 13–14 internodes; LD9, 15–16 internodes; LD10, 22–23 internodes; LD11, flowering; SD1, 2–3 rosette leaves; SD2, 4–5 rosette leaves; SD3, 6–7 rosette leaves; SD4, 8–9 rosette leaves; SD5, 10–11 rosette leaves; SD6, 12–13 rosette leaves; SD7, 1–2 internodes (just bolting); SD8, 3–4 internodes; SD9, 5–6 internodes; SD10, 7–8internodes; SD11, 9–10 internodes; SD12, 12–13 internodes; SD13, 16–17 internodes; SD14, 20–21 internodes. (**C**) Growth and development of *L. × formolongi* in different photoperiod. (**D**,**E**) The diurnal rhythm expression pattern of LfCRY2 under LDs and SDs. The shaded bars in each chart represent dark periods. (**F**) LfCRY2 transcript levels under different light quality during different irradiation time. Data points represent an average of three biological replicates with three technical replicates. Error bars represent the SD of three biological replicates.

*CRY2* gene expression was measured in *L. × formolongi* via quantitative reverse transcription polymerase chain reaction (RT-qPCR). The *LfCRY2* gene was constitutively expressed in all examined tissues (Figure 3A). Higher levels of expression of the *CRY2* gene were observed in the flower petals, which differed from other tissues, whereas lower levels of expression were detected in roots, bulbs, and stems (Figure 3A). 

*L. × formolongi* was a facultative LD plant, strongly affected by the photoperiodic flowering pathway [37]. To investigate whether the *LfCRY2* gene was involved in the lily photoperiodic flowering pathway, its mRNA expression levels were determined at 11 developmental stages under the LD condition and 14 developmental stages under the SD condition. The average number of rosette leaves and internodes at each stage was recorded as morphological markers. 

Under LDs, *LfCRY2* was highly expressed during the LD4 stage (8–9 leaves, just before bolting), LD9 stage (15–16 internodes), and LD11 stage (Figure 3B). The highest expression level was found at the LD4 stage (8–9 leaves, just before bolting), which has been identified as the floral induction phase [37]. Under SDs, the elongation of internodes continued for much longer than that under LDs (Figure 3B). Additionally, the expression of *LfCRY2* was significantly lower under SDs than under LDs throughout all stages (Figure 3B). The highest transcript accumulation appeared during the SD6 stage (12–13 leaves, just before bolting), and there was no remarkable transcript difference between different stages under SD conditions (Figure 3B). These results indicate that the *LfCRY2* gene may depend on photoperiod to promote flowering under LD conditions.

### 2.4. Circadian and Light Regulation of CRY2 Gene in L. × formolongi

To determine whether the photoperiod-dependent diurnal expression of *Arabidopsis CRY2* gene may be preserved in *L. × formolongi*, the diurnal expression profiles of *LfCRY2* in leaves of seedlings exposed to the LD and SD treatments were characterized by RT-qPCR at 19 time points over three days. Plant tissue was sampled just before bolting. Under LD, *LfCRY2* exhibited a clear circadian rhythm for three days, reaching its peak at dusk (Figure 3D). At dawn, *LfCRY2* showed low transcript level. Then, the expression of *LfCRY2* gradually increased during the last four hours of daylight and peaked at dusk (Figure 3D). At night, the transcript accumulation of *LfCRY2* gradually decreased (Figure 3D). Under SD, *LfCRY2* expression peaked 8 h after dark. During the three days, the expression pattern was different for each diurnal cycle (Figure 3E). Furthermore, it showed that *LfCRY2* may play important role in the photoperiodic flowering pathway under LD conditions in *L. × formolongi*. 

To explore whether the expression levels of *LfCRY2* were regulated by light, we measured the expression patterns of *LfCRY2* under different light conditions. During 48 h of continuous blue light irradiation, *LfCRY2* transcript accumulation showed dynamic changes. As shown in (Figure 3F), *LfCRY2* expression was high during the first 8 h of continuous blue light irradiation and peaked after 0.25, 2, and 8 h of irradiation, with the greatest peak after 2 h. However, the expression of *LfCRY2* decreased constantly upon irradiation with both white and red light, and the transcript accumulation was lower than that under blue light (Figure 3F). The expression level was lowest under red light irradiation. These results showed that the expression of *LfCRY2* may be induced by blue light and inhibited by red and white light.

### 2.5. Overexpression of LfCRY2 Promoted the Flowering of Arabidopsis under LDs

**Figure 4:**
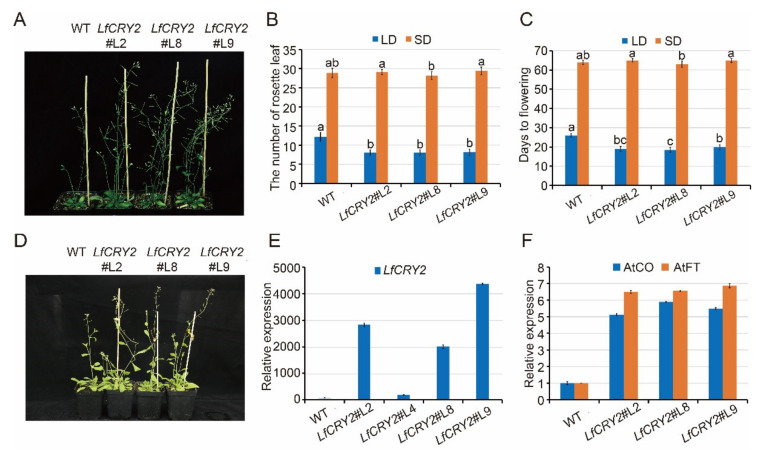
Overexpression of LfCRY2 promotes flowering in *Arabidopsis*. (**A**,**D**) Flowering traits of WT *Arabidopsis* and transgenic *35S::LfCRY2* lines under LDs and SDs. (**B**,**C**) The flowering time and rosette leaf number of WT *Arabidopsis* and transgenic *35S::LfCRY2* lines under different photoperiod. (**E**,**F**) Expression levels of *LfCRY2*, *AtCO*, and *AtFT* in WT *Arabidopsis* and transgenic *35S::LfCRY2* lines. Error bars represent the SD of three biological replicates.

To elucidate the function of *LfCRY2*, *LfCRY2* overexpression (*LfCRY2*-OE) *Arabidopsis* were generated using the floral-dip method. Among them, three independent homozygous *LfCRY2* T3 lines (L2, L8, L9) with relatively high expression levels were selected for further analysis (Figure 4A,E). To verify the involvement of *LfCRY2* in photoperiodic flowering, we analyzed the resulting flowering-time phenotype of wild-type (WT) and *LfCRY2*-OE seedlings grown under LD and SD conditions by measuring the days to flowering and the rosette leaf number at the bolting time. As demonstrated in Figure 4A, the transgenic seedlings exhibited a significantly earlier flowering phenotype on LDs compared with those of the controls (WT). The average days to flowering was 19 for transgenic seedlings, and 26 for WT seedlings (Figure 4B). The average number of rosette leaves was 8 for transgenic seedlings, and 12 for WT seedlings (Figure 4C). However, there was no difference between transgenic seedlings and WT seedlings under SD conditions (Figure 4B–D). The results indicated that *LfCRY2* had a promotional effect on photoperiodic flowering in *Arabidopsis* under LD conditions. Next, we tested the regulatory role of *LfCRY2* in the photoperiodic flowering pathway by analyzing the expression of *AtFT* and *AtCO* in the transgenic and WT seedlings under LD conditions. As shown in Figure 4F, the expression levels of *AtCO* and *AtFT* were upregulated considerably in transgenic seedlings compared to WT seedlings, which is consistent with the flowering phenotypes.

### 2.6. Overexpression of LfCRY2 Results in Longer Roots and Blue Light-Specific Hypocotyl in the Transgenic Arabidopsis

**Figure 5:**
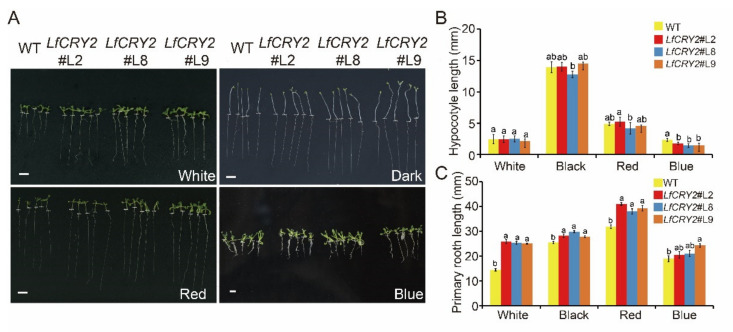
Comparison of the hypocotyl and root lengths of WT *Arabidopsis* and transgenic *35S::LfCRY2* lines. (**A**) Phenotypes of WT plants and three transgenic lines grown under darkness and blue, white, and red light conditions. The white dashed line indicates the boundary between hypocotyl and root. (**B**,**C**) Comparison of the hypocotyl and root lengths of WT plants and three transgenic lines grown under darkness and blue, white, and red light conditions. The scale bar represents 5 mm.

To determine the ability of *LfCRY2* to inhibit hypocotyl elongation, hypocotyl lengths of *LfCRY2*-OE and WT *Arabidopsis* seedlings were measured, respectively. As demonstrated in Figure 5A, the hypocotyl elongation of transgenic seedlings was repressed under blue light conditions, while there were no significant differences between the *LfCRY2*-OE seedlings and WT seedlings under dark, red light, and white light conditions (Figure 5B). Similar to *AtCRY2* [41], *LfCRY2* showed inhibition of hypocotyl elongation mediated by blue light in transgenic seedlings, indicating that the signaling of the *CRY2* gene in the inhibition of hypocotyl is conserved in *L.× formolongi*.

Interestingly, *LfCRY2* promoted root elongation in *Arabidopsis*. As shown in Figure 5C, the transgenic seedlings exhibited a lengthened root phenotype compared to WT seedlings not only under the dark, red light, and white light conditions, but also under blue light conditions, in contrast to *AtCRY2* [41]. The results demonstrated that *LfCRY2* promoted root elongation.

### 2.7. Subcelluar Localization

**Figure 6:**
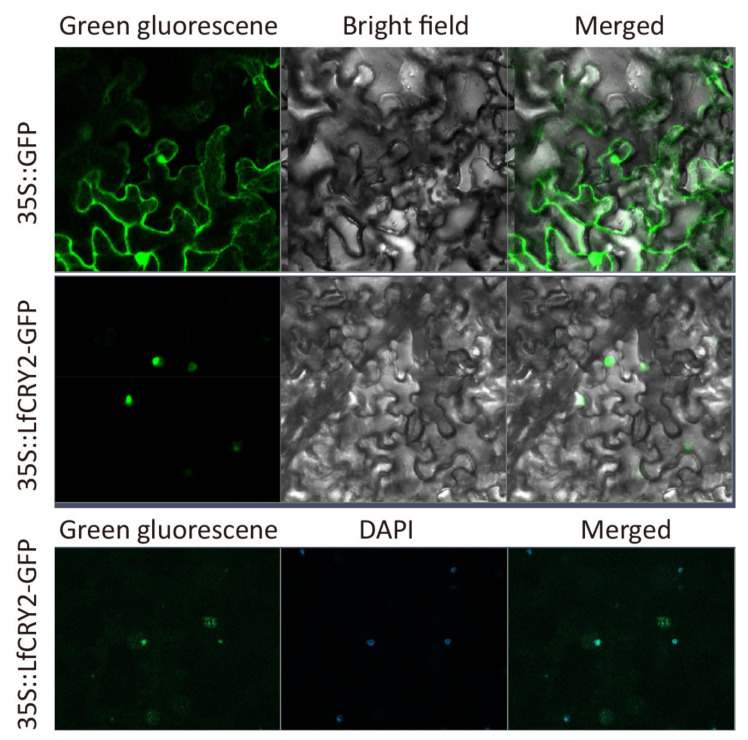
Subcellular localization of LfCRY2.

To investigate the sub-cellular localization of *LfCRY2*, the *LfCRY2* open reading frame (ORF) without the termination codon was fused upstream of the GFP reporter under the control of the *CaMV* 35S promoter, and the plasmid containing GFP alone was used as a control. The construct and GFP alone were introduced into epidermal cells of tobacco leaves and observed under a confocal microscope. As shown in Figure 6, the fusion protein has strong fluorescent signals in the nucleus. The results implied that the LfCRY2 protein was targeted to the nucleus.

### 2.8. LfCRY2 Interacts with LfCOL9 and AtCOB1 in Yeast and Plant Cells

**Figure 7:**
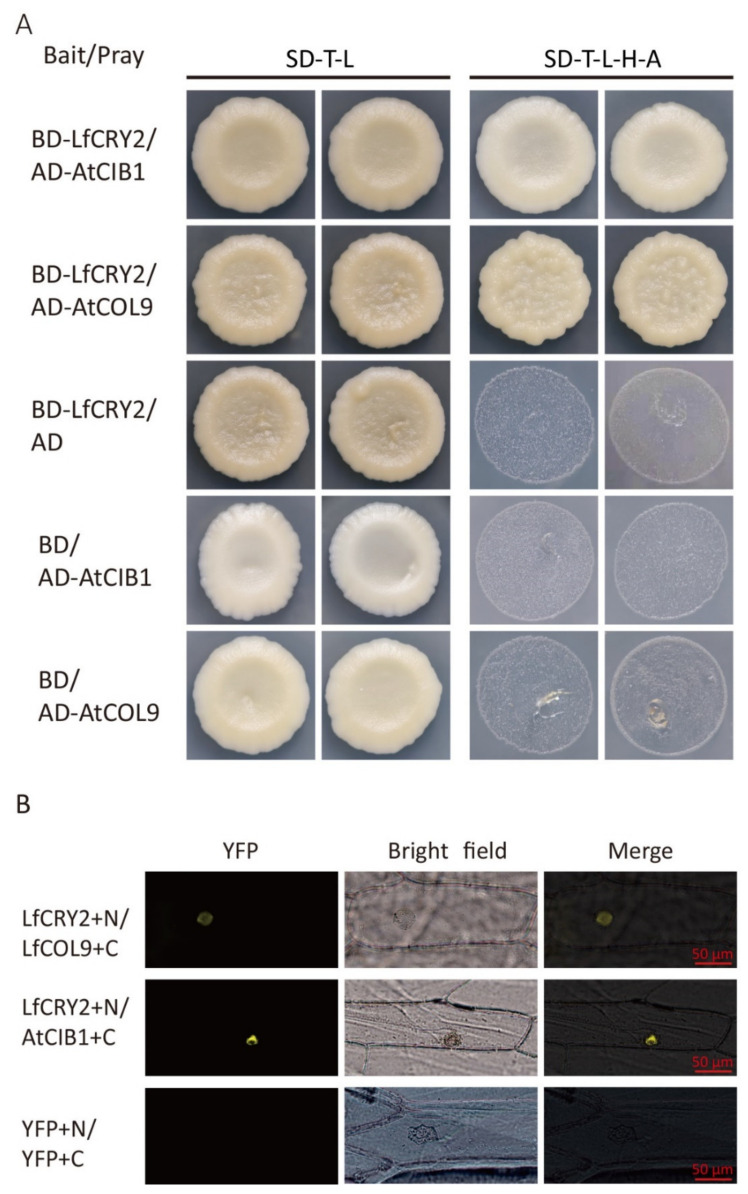
LfCRY2 physically interacts with LfCOL9 and AtCIB1 in yeast and onion cells. (**A**) Yeast two-hybrid showing the interaction of LfCRY2 with LfCOL9 and AtCIB1. Yeast cells coexpressing the indicated combinations of constructs were grown on nonselective (SD-T-L) or selective media (SD-T-L-H-A). (**B**) Bimolecular fluorescence complementation (BiFC) assays indicating the interaction of LfCRY1 with LfCOL9 and AtCIB1 in onion cells.

To determine whether the full-length LfCRY2 interacts with AtCIB1 or LfCOL9, GAL4 yeast two-hybrid assays were performed to investigate the protein–protein interactions by cotransforming yeast cells with a bait construct and a prey construct. After selecting synthetic defined media lacking threonine, leucine, histidine, and alanine (SD-TLHA), LfCRY2 was found to interact with AtCIB1 and LfCOL9, respectively, in yeast cells (Figure 7A). 

To further verify the interactions of LfCRY1 with AtCIB1 and LfCOL9, bimolecular fluorescence complementation (BiFC) assays were performed. The *LfCRY2* was tagged with the GFP N-terminal and *AtCIB1* or *LfCOL9* was tagged with the GFP C-terminal, then both fusion proteins were cotransformed in onion cells. Yellow fluorescence was observed in the nucleus when *LfCRY2* was coexpressed with *LfCOL9* and *AtCIB1*, suggesting that LfCRY2 can interact with both LfCOL9 and AtCIB1 in plant cells (Figure 7B). 

## 3. Discussion

To date, much of our knowledge regarding the function of CRYs in higher plants has been gained from studies in *Arabidopsis*. CRYs act as blue light receptors in the regulation of plant photomorphogenesis. In *Arabidopsis*, *CRY1* plays a major role in the control of the de-etiolation process while *CRY2* is important for flowering [12,13]. While *CRYs* are ubiquitous in vascular plants [42], relatively little is known about the function of *CRYs* in plant species other than *Arabidopsis*. In ornamental plants, there have not been extensive characterization except in *Chrysanthemum* [34] In this study, we identified one *CRY2* member from lilies and studied its expression patterns and biological functions, and preliminarily proved its function as a blue light receptor and flowering activator similar to that of *AtCRY2* in *Arabidopsis*.

CRY proteins typically consist of a well-conserved N-terminal PHR superfamily and a CCE domain independent of photolyase [14]. In *Arabidopsis*, the N-terminal PHR domain of CRYs primarily binds to the chromophore and is responsible for homodimer formation, while the CCE domain mainly acts as an effector to regulate signals through interactions with proteins [15,16,17,43]. The sequences of PHR, including the DNA photolyase domain and FAD-binding domain from different CRY orthologs, are strongly conserved. Similar to other CRY2 orthologs, the amino acid sequence of LfCRY2 contained a well-conserved DNA photolyase domain and FAD-binding domain (Figure 1A). Additionally, LfCRY2 included the TGYP motif conserved in all Type I photolyases (Figure 1B). Unlike the PHR domain, the CCE domain of CRYs varies greatly in length and sequence among different species, even among different CRY members in the same species. For example, the homology of the N-terminal PHR domain of *Arabidopsis* CRY1 and CRY2 is 58%, whereas the homology of the C-terminus is only 14% [44]. However, there are three CCE motifs conserved in most plants, called the DAS domain, which includes a DQXVP motif, an acidic motif region, and a STAESS motif. The DAS domain plays an important role in cell localization, intermolecular interactions, and physiological functions [45]. Although the length and similarity of the CCE domains among LfCRY2 were different to those of other orthologs, the two most prominent motifs (DQXVP and STAESS) in the C-terminal were conserved (Figure 1B,C). These analyses suggest that *LfCRY2* is an *AtCRY2* homolog that may have similar functions. The phylogenetic tree showed a clear boundary between dicotyledons and monocotyledons. *LfCRY2* was grouped into the monocotyledon group together with rice and wheat and showed the highest homology with *Musa acuminata CRY2* (Figure 2), which was consistent with plant evolution. 

Identifying the subcellular localization of proteins is important for understanding their function. Both *Arabidopsis* CRY1 and CRY2 are soluble nuclear proteins [45]. In this study, LfCRY2 was located in the nucleus, similar to AtCRY2, indicating that LfCRY2 may possess the same function as AtCRY2.

As blue light receptors, the expression of *CRYs* is regulated by light quality and photoperiod. The expression of *CRY* genes in *Arabidopsis* was induced by light, as expected, and exhibited a 24-h circadian rhythm [46]. Additionally, the *CRY* homologs in other species, such as in *Brassica* [47] and apples [48], are induced by blue light. In our light-treated experiments, we found that the transcript levels of *LfCRY2* were much higher in the blue light, which suggest that *LfCRY2* may be a blue light receptor in lilies. As a photoreceptor senses light signals directly, there is a high expression level of *CRY1* in leaves in most species since leaves are the organs that perceive light signals, as was found in sweet sorghum, *Chrysanthemum* and peas [33,34,49]. In this study, we found that *LfCRY2* were constitutively expressed in all the organs, similar to *AtCRY2* in *Arabidopsis*. However, *LfCRY2* showed higher expression levels in flower petals, which differed from other tissues (Figure 3A). The functions of *CRY* genes vary between different tissues [14]. The high expression of *LfCRY2* in petals suggests it has a flowering-related function in lilies. 

In the photoperiodic flowering pathway, *CRY2* is the primary photoreceptor that acts to induce flowering under LDs [13]. We previously demonstrated that *L. × formolongi* was a facultative LD plant strongly affected by the photoperiodic flowering pathway [37]. In the artificial climate chamber, 90% of *L. × formolongi* seedlings flowered within 36 weeks after sowing under LDs, while 30% of the seedlings flowered under SDs in this study (Figure 3C). To investigate the role of *LfCRY2* in the photoperiodic regulation of flowering, we analyzed the expression patterns in different developmental stages under different photoperiods. The transcript accumulation of *LfCRY2* under LDs was regulated in a photoperiod-dependent manner and peaked during the floral induction stage (just before and after bolting) (Figure 3B). Similarly, the highest transcription levels of the important flowering activator *LfCOL9* and florigen ortholog *LfFT1* occur in the floral induction stage [38]. These results demonstrate that *LfCRY2* may play an active role in photoperiodic flowering under LDs in lilies. 

The circadian clock is an intrinsic periodic system used to measure the duration of day and night periods in living organisms, and has profound impacts on many physiological processes in plants. Both phytochromes and cryptochromes entrain the circadian clock by mediating the input of day and night signals at dawn and dusk [50]. In *Arabidopsis*, *CRY1* and *CRY2* mRNA levels are regulated by the circadian clock, peaking towards the end of the light phase under a 12/12 h light/dark cycle [46]. Similarly, *LfCRY2* exhibited a circadian rhythm for three days under both LDs and SDs. Under LDs, the transcript level of *LfCRY2* accumulated rapidly during the four hours before dark and reached a broad peak at the end of the day (Figure 3D). The same pattern was observed in peas and soybeans. *PsCRY2b* expression in peas under LDs showed strong diurnal regulation, reaching a peak at the end of the day and falling during the night [49]; the *GmCRY1a* under SDs exhibited the same circadian rhythm in soybeans, a short-day (SD) plant [32]. Additionally, under SD conditions, the transcription level of *LfCRY2* peaked at approximately midnight or subjective midnight, since the SD time was lagging behind that of the LD conditions (Figure 3D,E). The circadian regulation of *LfCRY2* varies under different photoperiods, which is manifested by the accumulation and degradation of its transcriptional levels. The peak in the diurnal rhythm towards the end of the day is essential for stabilizing the CO protein and inducing flowering [51]. In *Arabidopsis*, *AtCRY2* and *AtCO* mRNA levels peak in the afternoon under LDs. AtCOP1 promotes the ubiquitin-mediated proteolysis of CO and the CRY-mediated signal that negatively regulates COP1, thereby stabilizing CO and activating *FT* transcription [52]. *LfCOL9*, the ortholog of *Arabidopsis CO* in lilies, also exhibited a diurnal peak at dusk [38]. Therefore, the peak in transcript level of *LfCRY2* at the end of the light phase under LDs may be important for LfCOL9 stability and floral induction. 

We examined the possible effects of *LfCRY2* on flowering time. It has been shown that *CRY2* plays a major role in flowering transition both in *Arabidopsis*, an LD plant, and rice, an SD plant [31,53]. For example, the *Arabidopsis cry2* mutant seedlings flower later than the wild-type seedlings under LDs but not SDs [13,54]. In the present study, the transgenic expression of *LfCRY2* in *LfCRY2*-OE seedlings accelerated flowering in *Arabidopsis* under LDs but not SDs (Figure 4A,B). These results further confirmed the promotional role of *LfCRY2* in photoperiodic flowering in response to LDs. Moreover, overexpression of *LfCRY2* promoted flowering in *Arabidopsis* by stimulating the mRNA expression of the *CO* and *FT*, in line with the phenotypic results. Hence, we inferred that *LfCRY2* could up-regulate *CO* expression, activating the *FT* expression that led to early flowering in transgenic *Arabidopsis*. 

In addition to stimulating flowering, *CRYs* regulate plant de-etiolation [18,35]. Photoinhibition of hypocotyl elongation is one of the most important features of de-etiolation. As blue light receptors, both *CRY1* and *CRY2* inhibit hypocotyl elongation in response to blue light in *Arabidopsis* and apples [35,36,48,55]. Similarly, we found that *LfCRY2* exhibited the same function as *AtCRY2* in the inhibition of hypocotyl elongation under blue light (Figure 5B). However, *LfCRY2* promoted root elongation regardless of light conditions (Figure 5C), contrary to *AtCRY2* and other *CRY2* orthologs [41]. Future research should explore the mechanism behind the root elongation effect of *LfCRY2*.

*CRY* genes participate in the photoperiodic regulation of flowering through several pathways. In one pathway, the photoactivated PHR domain of CRY2 interact with SPA1 and PHYA to suppress COP1-dependent degradation of CO, causing *FT* transcription and promoting photoperiodic flowering [15,25,26]. In another pathway, CRY2 binds directly to the bHLH transcription factor CIB1, a transcriptional activator that can bind to the *FT* promoter to promote its transcription and stimulate flowering [28]. CIB1 is the first blue light-dependent CRY2-interacting protein to be described that belongs to the 17-member bHLH subfamily [28,56]. CRY2, CIB1, and CO can form a protein complex that promotes *FT* expression in response to blue light [29]. Additionally, CRY2 interacts with the transcription factors TOE1 and TOE2 directly to promote their dissociation from CO, alleviating their inhibition of CO transcriptional activity and accelerating floral induction [30].

To date, the participation of the *CRY2* gene in the photoperiodic flowering pathway has been elucidated as described above. The factors involved include COP1, CIBs, SPAs, and TOEs [20,26,28,30,56]. We have demonstrated that LfCRY2 can interact with LfCOL9 and AtCIB1 via yeast two-hybrid and BiFC assays (Figure 7). Due to the large size of the *Lilium* genome, complete sequencing data is not yet available, and the homologous sequences of *COP1*, *CIB1*, *SPA1*, and other functional genes in *Lilium* remain unknown. The interaction of LfCRY2 with LfCOL9 and AtCIB1 supports the hypothesis that *LfCRY2* utilizes the CIB1-CO pathway to participate in photoperiodic flowering in a similar manner to *AtCRY2*. In *Arabidopsis*, there is crosstalk between CRY2-CO and CRY2-CIBs. CRY2, CIB1, and CO can form a protein complex that is regulated by the photoperiod and activates *FT* transcription in response to blue light [29]. Additionally, CRY2 was recruited to the FT chromatin by CIB1 and CO and all three proteins were bound to the same region within the FT promoter [29]. Whether the same crosstalk between the CRY2-CO and CRY2-CIBs occurs in the lily needs further verification. Furthermore, it is not known whether CRY2 participates in the CRY-COP1-SPA1 pathway. We isolated *AtCOP1* from *Arabidopsis* and analyzed the interaction between LfCRY2 and AtCOP1 in yeast two-hybrid assays. It was found that LfCRY2 did not interact with AtCOP1 (results not shown). To further understand the molecular mechanisms underlying the involvement of *LfCRY2* in photoperiodic regulation of flowering, it is important to obtain the homologous sequences of COP1, SPA1, and PHYA in *L. × formolongi*. Further investigation is needed to identify other proteins that can interact with LfCRY2 and to determine how these interactions regulate flowering. 

In summary, we provide evidence that *LfCRY2* plays a vital role in the promotion of photoperiodic flowering under LDs. Expression analysis indicated that *LfCRY2* mRNA was induced by blue light, exhibited high expression levels during the floral induction stage, and demonstrated a circadian rhythm. In the investigation of *LfCRY2*-OE seedlings, it was found that *LfCRY2* played a role in de-etiolation under blue light and photoperiodic flowering under LDs. The early flowering phenotype of *LfCRY2*-OE seedlings were attributed to the increased expression of the critical regulatory module *CO/FT* related to flowering time. Additionally, LfCRY2 interacted with LfCOL9 and AtCIB1, supporting the hypothesis that *LfCRY2* hastens the floral transition via the CIB1-CO pathway in a similar manner to *AtCRY2*. A valuable feature of *L. × formolongi* is that flowering occurs within a year of seed propagation, and floral induction is very sensitive to photoperiod. This study contributes to a greater understanding of the function of *LfCRY2* in the photoperiodic flowering pathway. Understanding the molecular mechanisms of photoperiodic flowering is important to understand annual flowering in *L. × formolongi*. The identification of the functional genes involved in floral induction, such as *LfCRY2*, is also beneficial for the molecular breeding of lilies with shorter vegetative stages.

## 4. Materials and Methods

### 4.1. Phylogenetic and Bioinformatic Analyses

The Simple Modular Architecture Research Tool (SMART) database (http://smart.embl-heidelberg.de accessed on 31 October 2021) was used to analyze conserved domains and the SIB Expasy Bioformatics Resources Portal (http://expasy.org/accessed on 31 October 2021) was used to determine the protein properties of LfCRY2. The conserved motif logos were generated by the Weblogo program using default parameters (http://weblogo.berkeley.edu/logo.cgi accessed on 31 October 2021). Amino-acid sequences of CRY2 homologs were downloaded from the National Center for Biotechnology Information (NCBI) website (https://www.ncbi.nlm.nih.gov accessed on 31 October 2021) and aligned with LfCRY2 using the ClustalW2.1 program (http://www.genome.jp/tools/clustalw accessed on 31 October 2021). Multiple sequence alignments were performed using DNAMAN 7.0 software. A phylogenetic tree was built by MEGA 11.0 software using the neighbor-joining method.

### 4.2. Plant Materials and Growth Conditions

Fifteen *L. × formolongi* cv. Raizan 2 seedlings were grown in incubators under LD (light/dark: 16/8 h) and SD (light/dark: 8/16 h) conditions at a thermoperiod of 25/18 °C (day/night). The seedlings initially developed rosette leaves for several months, then bolted and entered the internodal growth stage. The number of rosette leaves and internodes were monitored weekly. Bolting time, flowering time, and flowering rate were recorded. 

### 4.3. Expression of LfCRY2 Gene

For the tissue-specific assay, samples of roots, bulbs, leaves (from the middle of the stem), stems, and flower petals of *L. × formolongi* were collected during the flowering stage under LDs. Additionally, to assess the changes in *LfCRY2* expression during different developmental stages under different photoperiods, leaves were sampled every week after seed germination. For the diurnal expression assay, seedlings from both photoperiods were sampled every 4 h for three days during the floral induction phase (i.e., just before bolting) [37]. For the light treatment, seedlings in the floral induction phase were grown in the dark for seven days and then placed under blue light (13.0 µmol m^−2^ s^−1^), red light (22.3 µmol m^−2^ s^−1^) or white light (17.0 µmol m^−2^ s^−1^) for 24 h. Leaves were sampled at 0, 0.25, 0.5, 1, 2, 4, 8, 12, and 24 h after the treatment. All samples were collected from three biological replicates at each time point and immediately frozen in liquid N and stored at −80 °C until further analysis.

Quantitative real-time PCR (RT-qPCR) was performed using SYBR^®^ Green Master mix (Takara Bio Inc., Dalian, China) on a CFX96 Real-Time PCR Detection System (Bio-Rad, California, USA) according to the manufacturer’s instructions. *L. × formolongi* Aquaporin *TIP4-1* was selected as a reference gene to standardize the results [57]. All primers used in the analysis are listed in Table S1. Primer efficiency was calculated before performing RT-qPCR. The relative gene expression levels were calculated using the 2^−ΔΔC^_T_ method [58].

### 4.4. Arabidopsis Transformation and Functional Analysis

#### 4.4.1. Arabidopsis Transformation

DNA fragments containing the full-length coding sequence of *LfCRY2* were PCR-amplified from the cDNA of *L. × formolongi* using gene-specific primers (Table S1). To generate transgenic plants harboring the *LfCRY2* gene, the full-length coding sequence of *LfCRY2* were amplified and subcloned into the *Sal*I and *Kpn*I-digested pCAMBIA1300 (GenBank No. AF234297) vector using the Trelief^TM^ SoSoo Cloning Kit (TsingKe Company, Beijing, China). The recombinant vectors were subsequently introduced into the *Agrobacterium tumefaciens* strain GV3101 by the freeze–thaw method [59]. Transformation of *Arabidopsis* Col-0 was performed by the floral-dip method [60]. Transgenic plants were selected on Murashige and Skoog (MS) agar plates [38] containing 25 mg.L^−1^ of hygromycin and 250 mg.L^−1^ of cefotaxime, and gene insertion was confirmed by RT-PCR using the gene-specific primers. The homozygous T3 lines were used for further assays.

#### 4.4.2. Flowering Studies

The *Arabidopsis* (Col-0 wild-type and T3 seedlings of Col-0 wild-type overexpressing *LfCRY2*) seedlings were germinated and grown separately in an incubator under LDs (16 light/8 h dark) and SDs (8 light/16 h dark). Flowering time was measured as the number of days from sowing to flowering and the number of rosette leaves at bolting, from three *LfCRY2*-OE lines under both LDs and SDs, respectively. At least 20 seedlings were analyzed. Rosette leaves of Col-0 wild-type and transgenic seedlings were sampled when the *LfCRY2*-OE lines just bolting to analyze the transcript levels of *AtCO* and *AtFT* by RT-qPCR. *Arabidopsis* TUBULIN BETA-2 CHAIN (*TUB2*) was selected as a reference gene to standardize the results. All primers are listed in Table S1. 

For hypocotyl and primary-root phenotype analysis, seeds of Col-0 wild-type and transgenic lines were germinated in MS medium and irradiated (light/dark: 8/16 h) with white, blue, or red light or kept in the dark in an incubator at 22 °C. Hypocotyl lengths and primary root lengths of at least 20 seedlings grown in described conditions were measured after 8 days of growth. These experiments were performed in at least three independent biological repetitions.

### 4.5. Subcellular Localization

To generate the plant green fluorescent protein (GFP) expression vector *35S::LfCRY2-GFP*, the coding sequence of *LfCRY2* without the stop codon was amplified and inserted into the *Bam*HI site of the pBI121-GFP vector using the SoSoo Cloning Kit (TsingKe Company, Beijing, China). The recombinant vector was introduced into the *Agrobacterium* strain GV3101. *Agrobacterium*-mediated transient expression in young leaves of *Nicotiana benthamiana* was performed. Nuclei staining was done using 4′,6-diamidino-2-phenylindole (DAPI). Subcellular localization of the LfCRY2-GFP fusion protein was determined using confocal laser scanning microscopy at 72 h after infection (CarlZeiss LSM710, Oberkochen, Germany). 

### 4.6. Yeast Two-Hybrid Assay

The GAL4 yeast two-hybrid systems were made with the vectors pGBKT7 and pGADT7, which carry the tryptophan and leucine selection marker, respectively. The coding sequences of *AtCIB1* and *LfCOL9* were PCR-amplified using gene-specific primers (Table S1) and cloned into the pray vectors pGADT7 (AD). The full-length *LfCRY2* cDNA was cloned into the bait vector pGBKT7 (BD). GAL4 yeast two-hybrid assays were performed in accordance with the manufacturer’s instructions (Matchmaker user’s manual; Mountain View, CA, Clontech). BD-LfCRY2, AD-LfCOL9, and AD-AtCIB1 cassettes were cotransformed into the AH109 yeast strain via the polyethylene glycol/lithium acetate (PEG/LiAc) transformation procedure. Transferred yeast cells were then spread onto SD-TLHA. Transformed colonies were plated onto SD-TLHA and dyed with X-gal to test for possible interactions.

### 4.7. Bimolecular Fluorescence Complementation (BiFC) Assay

BiFC constructs were built with the vectors pSPYCE-35S and pSPYNE-35S, which carry fragments encoding the carbon (C)- and N-terminal halves of GFP (cGFP and nGFP respectively). The coding sequence of *AtCIB1* and *LfCOL9* without the stop codon was amplified and inserted into the destination vector pSPYCE-35S (cGFP fused to the N-terminals of the genes), generating *35S::AtCIB1-cGFP* and *35S::LfCOL9-cGFP*, respectively. The full-length *LfCRY2* cDNA was cloned into pSPYNE-35S (nGFP fused to the C-terminus of the genes) to generate *35S::LfCRY2-nGFP*. All the combinations were introduced into the *Agrobacterium* strain GV3101. The *Agrobacterium*-harboring constructs expressing nGFP and cGFP fusion proteins were mixed at a ratio of 1:1 and were infiltrated into onion cells. After incubation at 22–23 °C for three days, the GFP-dependent fluorescence in the lower epidermal cells of leaves was detected using the confocal laser scanning microscope (CarlZeiss LSM710, Oberkochen, Germany). Each BiFC assay was performed with at least three independent plants with two to three leaves infiltrated for each, and one representative result is shown. 

All the statistical analyses in this paper were conducted in SPSS (Statistical Product and Service Solutions) software using Duncan statistical tests. Statistical significance was defined as *p* < 0.05.

## Supplementary Materials

The following are available online at https://www.mdpi.com/article/10.3390/ijms222312929/s1.
