# Supplementary material for: Cryptochrome 2 from Lilium × formolongi Regulates Photoperiodic Flowering in Transgenic Arabidopsis thaliana
Source: Int J Mol Sci. 2021 Nov 29;22(23):12929. doi: 10.3390/ijms222312929 (PMC8657805; doi:10.3390/ijms222312929)
Supplement: Supplementary file 1 [file ijms-22-12929-s001.zip › Supplementary materials.pdf]

**Table S1. Primers used in PCR analysis.**

| primer | sequence |
|---|---|
| Clone-LfCRY2-F | ATGGGTTCCCAATTGAAGACTA |
| Clone-LfCRY2-R | TTAGGGTTCGGCAGGC |
| Clone-LfCOL9-F | ATGGGGTCAGTTTGTGATTTC |
| Clone-LfCOL9-R | TCAATAGCTTCTTGTCTGGCAG |
| Clone-AtCIB1-F | ATGAATGGAGCTATAGGAGG |
| Clone-AtCIB1-R | CTTAATCAAACTCCTA |
| qPCR-LfCRY2-F | TACTCAAAGCTGCCGGGGTT |
| qPCR-LfCRY2-R | GCATTATCCAGCTCCCACAT |
| qPCR-LfEF-F | GGCACTAACTCGCTCCTTCTG |
| qPCR-LfEF-R | TTGGTAAGATGCTGGTGATTGGAT |
| qPCR-AtCO-F | TTGCTTCGTGGCTGTTCCCTAATTC |
| qPCR-AtCO-R | TCTATCTCCCCCGTAGCTCGTCTGT |
| qPCR-AtFT-F | TGGTGGAGAAGACCTCAGGAACT |
| qPCR-AtFT-R | TCATTGCCAAAGGTTGTTCCAG |
| qPCR-AtTUB2-F | ATCCGTGAAGAGTACCCAGAT |
| qPCR-AtTUB2-R | AAGAACCATGCACTCATCAGC |
